# Characterisation of macrophage infiltration and polarisation based on integrated transcriptomic and histological analyses in Primary Sjögren’s syndrome

**DOI:** 10.3389/fimmu.2023.1292146

**Published:** 2023-11-03

**Authors:** Yuan Zong, Yi Yang, Jiawen Zhao, Lei Li, Danyang Luo, Jiawei Hu, Yiming Gao, Li Wei, Ning Li, Liting Jiang

**Affiliations:** ^1^ Department of Stomatology, Ruijin Hospital, Shanghai Jiao Tong University School of Medicine, College of Stomatology, Shanghai Jiao Tong University, Shanghai, China; ^2^ Department of Pathology, Ruijin Hospital, Shanghai Jiao Tong University School of Medicine, Shanghai, China; ^3^ Department of Orthopaedics, Shanghai Key Laboratory for Prevention and Treatment of Bone and Joint Diseases, Shanghai Institute of Traumatology and Orthopaedics, Ruijin Hospital, Shanghai Jiao Tong University School of Medicine, Shanghai, China

**Keywords:** Sjogren’ s syndrome, immune microenvironment, macrophage, senescence-associated secreted phenotype (SASP), single-cell RNA analysis

## Abstract

**Background:**

Primary Sjögren’s syndrome (pSS) is a progressive inflammatory autoimmune disease. Immune cell infiltration into glandular lobules and ducts and glandular destruction are the pathophysiological hallmarks of pSS. Macrophages are one of the most important cells involved in the induction and regulation of an inflammatory microenvironment. Although studies have reported that an abnormal tissue microenvironment alters the metabolic reprogramming and polarisation status of macrophages, the mechanisms driving macrophage infiltration and polarisation in pSS remain unclear.

**Methods:**

Immune cell subsets were characterised using the single-cell RNA sequencing (scRNA-seq) data of peripheral blood mononuclear cells (PBMCs) from patients with pSS (n = 5) and healthy individuals (n = 5) in a public dataset. To evaluate macrophage infiltration and polarisation in target tissues, labial salivary gland biopsy tissues were subjected to histological staining and bulk RNA-seq (pSS samples, n = 24; non-pSS samples, n = 12). RNA-seq data were analysed for the construction of macrophage co-expression modules, enrichment of biological processes and deconvolution-based screening of immune cell types.

**Results:**

Detailed mapping of PBMCs using scRNA-seq revealed five major immune cell subsets in pSS, namely, T cells, B cells, natural killer (NK) cells, dendritic cells (DCs) and monocyte-macrophages. The monocyte-macrophage subset was large and had strong inflammatory gene signatures. This subset was found to play an important role in the generation of reactive oxygen species and communicate with other innate and adaptive immune cells. Histological staining revealed that the number of tissue-resident macrophages was high in damaged glandular tissues, with the cells persistently surrounding the tissues. Analysis of RNA-seq data using multiple algorithms demonstrated that the high abundance of pro-inflammatory M1 macrophages was accompanied by the high abundance of other infiltrating immune cells, senescence-associated secretory phenotype and evident metabolic reprogramming.

**Conclusion:**

Macrophages are among the most abundant innate immune cells in PBMCs and glandular tissues in patients with pSS. A bidirectional relationship exists between macrophage polarisation and the inflammatory microenvironment, which may serve as a therapeutic target for pSS.

## Introduction

Primary Sjögren’s syndrome (pSS) is an autoimmune disease characterised by ocular and oral dryness (1). As the second most prevalent autoimmune disease, pSS predominantly affects middle-aged women, with the estimated prevalence being approximately 43.03 per 100,000 habitants (2, 3). At present, minor salivary gland biopsy is considered the gold-standard strategy for diagnosing pSS (4). The main pathological feature of pSS is the lymphocytic infiltration of exocrine glands, especially salivary and lachrymal glands, leading to glandular atrophy and hypofunction. However, some patients may present with extra-glandular symptoms (5). Another important pathological feature of pSS is its close relationship with the presence of autoantibodies, particularly anti-SS-related antigen A (SSA) and anti-SSB antibodies (6). The aberrant distribution of anti-Ro/SSA and anti-Ro/SSB autoantibodies is a common occurrence in the cytoplasm of epithelial cells in patients with pSS (7); however, the pathological mechanism of pSS remains unclear. Therefore, understanding the molecular mechanisms underlying the pathogenesis of pSS is necessary for its early diagnosis and prompt treatment.

Numerous factors, including genetic, environmental and hormonal factors; B-cell hyperreactivity and epithelial activation have been associated with the pathogenesis of pSS (8, 9). In addition, recent studies have reported that pSS is associated with the disruption of salivary gland and immune homeostasis (10). Although the degree of T-lymphocyte infiltration is considerably high in minor salivary gland lesions, it cannot be inferred that pSS is dominated by T cells (7). The composition of lymphocytes infiltrating the periductal areas varies based on the severity of lesions. T cells play a pivotal role in mild lesions, whereas B cells predominate in severe lesions. Upon recruitment, various immune cells, including dendritic cells (DCs), macrophages and lymphocytes, interact with the salivary gland epithelium and enhance the inflammatory response (7, 11). The pathogenesis of pSS involves three major steps. The innate immune system, specifically Toll-like receptors (TLRs), serves as the first line of defence against pathogens. The activators of innate immunity trigger the activation of epithelial cells and DCs and the subsequent release of interferons (IFNs), which further promote B-cell activation (12). Fully activated macrophages, monocytes and DCs produce interleukin-12 (IL-12), which is essential for T-cell activation. The IL-12–IFN-γ axis is considered to be involved in the third step of pSS pathogenesis (3, 9). In recent years, innate immune cells have attracted substantial interest in the development of therapeutic strategies targeting the immune response.

Macrophages are multi-functional innate immune cells that orchestrate tissue repair, immune responses and inflammation (13). First described by Elie Metchnikoff, macrophages are derived from embryonic haematopoietic progenitors or monocytes and differentiate into tissue macrophages after entering the tissue (14–16). Because macrophages play an indispensable role in maintaining systemic homeostasis and participating in immune defence against pathogens and cellular debris, they are widely distributed across various tissues, including the salivary glands (15, 17). Recent studies have shown that macrophages perform several other functions in addition to phagocytosis (15). Macrophages and T cells can collectively regulate inflammation and participate in various functions such as phagocytosis and antigen presentation. This phenomenon has been partly confirmed in a noninflamed murine submandibular salivary gland model, as macrophages facilitate the local accumulation of tissue-resident memory CD8^+^ T cells (T^+^_RM_) at the site of inflammation (18). Tissue-resident immune cells are thought to be complemented by salivary gland progenitor cells (SGPCs) for the maintenance of normal salivary gland homeostasis (10). Upon tissue damage, epithelial cells release inflammatory factors and recruit macrophages to induce inflammatory responses. Through their uncontrolled and rapid cytokine production, macrophages recruit more immune cells to amplify the local inflammatory response, thereby bridging innate and adaptive immunity.

Recent studies have reported that macrophages contribute to the onset or development of pSS (7, 11). In addition to producing CCL22 that enhances IFN-γ production by T cells (19), macrophages can recruit lymphocytes and participate in co-stimulation through TLRs (3). The serum level of macrophage migration inhibitory factor (MIF) is reported to be high in patients with pSS (20). Therefore, macrophages may act as an important diagnostic and therapeutic target for pSS, and their role in the development of pSS warrants further investigation.

In this study, we analysed the single-cell transcriptomic data of PBMCs from healthy individuals and patients with pSS in a GEO dataset to construct a landscape of immune cells and examine the glandular immunological features. The abundance of monocyte-macrophages was higher in patients with pSS than in healthy individuals, with the cells being involved in different biological processes in the two groups. Furthermore, we isolated tissue-resident macrophages from labial salivary gland (LSG) biopsy samples collected from patients with pSS. These samples were subjected to RNA-seq and histological staining, revealing that the interaction between macrophages and the inflammatory microenvironment is a potential mechanism underlying the pathogenesis of pSS.

## Materials and methods

### Patients and human tissue samples

All human tissues were obtained with the approval by the Ethics Committee of Ruijin Hospital, Shanghai Jiao Tong University School of Medicine and the Chinese Clinical Trial Registry (ChiCTR2000039820). The final study cohort consisted of 24 patients with pSS 35 to 60 years of age and 12 controls with non-pSS who were age and sex matched. The 2016 American College of ACR/EULAR classification were used to provide a valid diagnostic criterion of pSS (21). Those who had xerostomia or xerophthalmia but did not match the classification criteria for pSS were classified as non-pSS individuals. Before collecting LSG samples and clinical data, each participant completed an informed consent form. At the time of the LSG biopsy, there was no history of the patient receiving immunosuppressive or steroid therapy.

### Tissue processing and immunohistochemistry staining

Fresh LSG samples were fixed in 10% neutral buffered formalin for an overnight period at room temperature and then processed for paraffin embedding. Paraffin-embedded tissue sections (4μm) were first air-dried and then dried at 75°C for 2 hours. Hematoxylin and eosin (H&E) standard staining was performed using a CoverStainer (Dako, Germany) following the manufacturer’s procedures. Immunohistochemical procedures were performed automatically on Leica Bond RX automated staining platform (Leica Biosystems, Welzlar, Germany using a BOND Polymer Refine Detection kit (DS9800, Leica Biosystems). Primary antibodies used in the current study included the following: CD68 (GA613, Dako, Denmark), BCL2 (IR614, Dako, Denmark), CK7 (IR619, DAKO, Denmark), Cytochrome c (1:3000, ab133504, Abcam, USA), Cytochrome c (1:3000, 10993-1-AP, Proteintech, China), MMP9 (1:200, 10375-2-AP, Proteintech, China), TNF-α (1:1000, 60291, Proteintech, China), P16 (Roche, Switzerland) and P53 (IR616, DAKO, Denmark). Images were photographed with a microscope (Nikon Eclipse Ni-U) equipped with a digital camera (Nikon DS-Ri).

### Immunofluorescence staining and confocal imaging

Multiple fluorescence staining was performed on LSG tissue samples as described previously (22). Primary antibodies were as follows (dilutions are indicated in parentheses): CD68 (1:200, 28058-1-AP, Proteintech, China), CD3 (1:200, 17617-1-AP, Proteintech, China), CD20 (1:100, ab64088, Abcam, USA), CD31 (1:200, 11265-1-AP, Proteintech, China), VWF (1:50, 27186-1-AP, Proteintech, China) and CK7 (1:200, 17531-1-AP, Proteintech, China). Cell nuclei were labelled with DAPI. A TCS SP8 MP confocal microscope (Leica, Wetzlar, Germany) Images was used to photograph representative fluorescence samples.

### Data collection and single-cell RNA-seq data processing

The single-cell RNA-sequencing data of peripheral blood mononuclear cells (PBMCs) from 5 healthy individuals and 5 patients with pSS were extracted from the Gene Expression Omnibus database (GSE157278 dataset) (https://www.ncbi.nlm.nih.gov/geo/). The Seurat R package (http://satijalab.org/seurat/) was used for quality control and downstream analysis. Cells with nCount_RNA of <1000 or >60,000; nFeature_RNA of <500 or >6,000 and cells with a percentage of mitochondrial genes >20% were filtered out. After quality control, 33,694 genes were detected in 56,345 cells, and t-SNE was used to visualise cell clusters (23). Each dot corresponded to a single cell in the t-SNE plot, and the clusters were manually annotated based on well-known immune cell markers using SingleR (24). The following genes were used for cell type annotation: CD3D, CD3E, CD3G and CD4 for CD4^+^ T cells; FOXP3 for Treg cells; CD8A and CD8B for CD8^+^ T cells; IGHD for naïve B cells; MS4A1, CD79A and CD27 for memory B cells; CD14 for monocytes; CD68 for macrophages; CD1C for DCs; MZB1 for platelets; ALDH1A1 and PROM1 for endothelial cells and KLRF1 and CD247 for NK cells. To characterise monocyte-macrophage subpopulations in PBMCs, the curated monocyte-macrophage data were further subjected to dimensionality reduction and clustering. Gene set enrichment analysis (GSEA) was used to screen for enriched genes in the Hallmark and GO gene sets in each cluster.

### Bulk RNA sequencing

Bulk RNA Sequencing was performed on fresh LSG samples as described previously (22). Briefly, total RNA was extracted from LSG biopsy samples using the TRIzol reagent (Invitrogen, USA) and then quantified using an Agilent 2100 bioanalyser (Agilent Technologies, CA, USA) and a NanoDrop spectrophotometer (Thermo Fisher Scientific Inc.). All libraries were sequenced on an Illumina Novaseq system (Illumina, CA, USA). Sequencing was done in a 2x 150 paired-end (PE) configuration. Raw sequencing reads were aligned to the human reference genome GRCh38 using software Hisat2 (v2.0.1).

### Weighted gene co-expression network analysis

Clinical samples were divided into high- and low-M1-macrophage-infiltration groups, with 18 samples in each group. The WGCNA R package was used to establish a co-expression network to identify modules with highly correlated genes (25). The modules were visualised using an organic layout in Cytoscape (26). The DAVID (https://david.ncifcrf.gov/) and Metascape (http://metascape.org) (27) tools were used to implement functional annotation analysis. Based on RNA-sequencing data, hub genes associated with M1 macrophage infiltration were identified and subjected to KEGG pathway analysis as defined in Proteomaps (www.proteomaps.net) (28).

### Gene set enrichment analysis

KEGG, Hallmark and WIKI-pathway gene sets were used for GSEA (https://www.gsea-msigdb.org/gsea/). All differentially expressed pathways with a normalised (NOM) P-value of <0.05 were selected for subsequent analysis. The ggplot2 R package (http://ggplot2.tidyverse.org/) was used to generate heat maps based on the fragments per kilobase of transcript per million sequenced reads (FPKM) data of hub genes expressed in minor salivary gland tissues. A list of hub mitochondria-related pathways was obtained from the Mitocarta 3.0 database (29). Subsequently, the single-sample gene set enrichment analysis (ssGSEA) algorithm (30) was used to calculate the enrichment score of each pathway. The ‘pheatmap’ R package was used to examine the correlation between mitochondria-related pathways and glandular microenvironment genes (Spearman correlation analysis). Interacting macrophage-related genes were predicted by using GeneMANIA (https://genemania.org/).

### Analysis of immune cell infiltration and the glandular microenvironment

Marker genes reported in previous studies and the CellMarker database (31) were used to annotate each cell subgroup (47 immune cells and 8 salivary glandular cells) based on RNA-seq data. The overall immune and stromal cell infiltration in LSG samples was evaluated using the ESTIMATE package (32). The CIBERSORT (http://cibersort.stanford.edu) and ssGSEA algorithms were used to evaluate the proportion of immune cells in LSG samples from patients with pSS. The correlation between different immune cells was examined using the corrplot R package (Pearson correlation analysis). Subsequently, the correlation between macrophages and other immune cell types was examined using the ggstatsplot package, and heat maps were generated using the ggplot2 package.

### Statistical analysis

All data were expressed as the mean ± standard deviation (SD). Differences between groups were estimated using Student’s t -test. The GraphPad Prism software (GraphPad) was used for statistical analysis. Statistical significance was denoted as follows: *, p < 0.05; **, p < 0.01; ***, p < 0.001.

## Results

### Peripheral blood immune cell landscape of pSS based on unsupervised clustering

**Figure 1:**
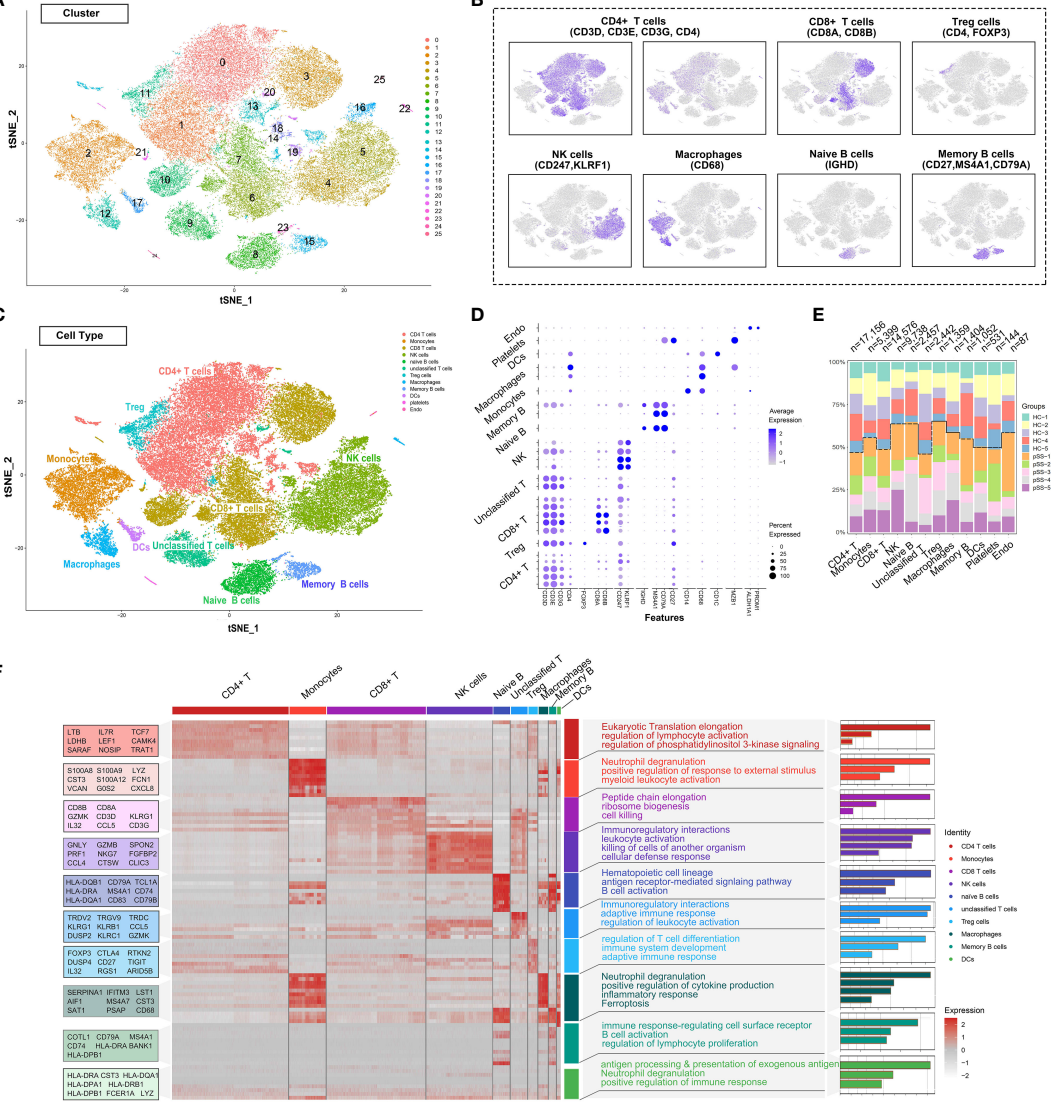
Deep profiling of the immune microenvironment based on scRNA-seq data of PBMCs from patients with pSS in a public dataset. **(A)** t-distributed stochastic neighbour embedding (t-SNE) plots demonstrating the single-cell transcriptomes of 56,345 cells. Unsupervised clustering revealed cellular heterogeneity, with 26 distinct cell clusters (colour-coded in legends). **(B)** Feature plots demonstrating the expression of key genes used for manual annotation of immune cell clusters on the t-SNE plot. **(C)** Dimensionality reduction using t-SNE; 12 main cell types were manually annotated in PBMCs. **(D)** Dot plot representing the percentage and average expression of selected marker genes in each cell cluster. **(E)** Bar plot depicting the proportion of each cell subset in each sample. **(F)** Heat map depicting the expression of the top 10 upregulated genes identified in each cell cluster. Each row represents a single cell, and each column represents a single gene. This list was further analysed using Metascape (metascape.org) to identify all statistically enriched GO terms. t-SNE, t-distributed stochastic neighbour embedding; PBMCs, peripheral blood mononuclear cells.

The single-cell RNA-sequencing data of PBMCs from patients with pSS and healthy individuals were extracted from the GSE157278 dataset. Immune cell populations in PBMCs were annotated, followed by graph-based clustering and dimensionality reduction using the t-SNE algorithm for visualisation of results. After rigorous quality control, 25 distinct PBMC clusters were obtained from 56,345 cells (collected from 10 patients) (**Figure 1A**). Canonical and cluster-specific marker genes were used to annotate the clusters to their specific cell types (**Figure 1B**). A total of 12 major cell types were identified from the 25 clusters as follows: CD4^+^ T cells, Treg cells, CD8^+^ T cells, unclassified T cells, natural killer (NK) cells, naïve B cells, memory B cells, monocytes, macrophages, DCs, platelets and endothelial cells (**Figure 1C**). The marker genes exhibited high expression in corresponding cell types, such as foxp3 in Treg cells and CD247 and KLRF1 in NK cells (**Figure 1D**). Additionally, our analysis showed that TRDV2 was the most significant gene upregulated in unclassified T cells (**Supplementary Table 1** **),** as the gene encoded by TRDV2 was referred to as Vδ2 TCR chains. Considering that unclassified T cells shared the CD4 T cell signature, and expressed γδ T cell genes, it may be a new subtype candidate. Subsequently, we compared the abundance of different cell types in PBMCs. CD4^+^ T cells had the largest population, with a total of 17,156 cells in all samples, whereas CD8^+^ T cells had the second largest population, with a total of 3,500 cells in all samples (**Figure 1E**). Accompanying the clustering, we also listed the top 10 significantly changed genes as features to cluster and analysed the functional enrichment of each cluster. The results showed that CD4^+^ T cells were associated with eukaryotic translation elongation and macrophages were associated with neutrophil degranulation and myeloid leukocyte activation (**Figure 1F**).

### High abundance of monocyte-macrophages in PBMCs from patients with pSS

**Figure 2:**
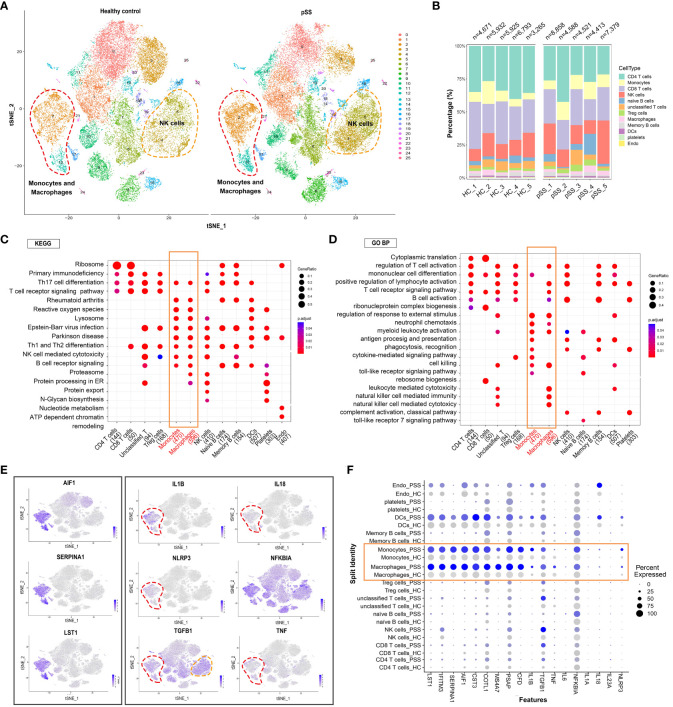
Analysis of immune cell subpopulations and gene expression and GO/KEGG pathway analysis of PBMCs from healthy individuals and patients with pSS. **(A)** t-SNE plot demonstrating innate and adaptive immune cells in PBMCs from healthy individuals and patients with pSS. **(B)** Bar plot depicting the proportion of each cell type in PBMCs from healthy individuals (n = 5) and patients with pSS (n = 5). **(C)** KEGG analysis revealed the specific metabolic pathways enriched in each immune cell cluster. **(D)** Gene Ontology (GO) analysis revealed biological processes associated with each immune cell cluster. **(E)** t-SNE feature plots demonstrating the relative expression of differentially expressed genes (AIF1, SERPINA1 and LST1) in monocyte-macrophages and inflammation-associated genes (IL1B, IL18, NLRP3, NFKBIA, TGFB1 and TNF) in each cluster. **(F)** Violin plots demonstrating the expression of differentially expressed genes in monocyte-macrophages and inflammation-associated genes in each cluster in patients with pSS versus healthy individuals.

t-SNE revealed that the distribution of different immune cell subsets in PBMCs varied between the control and pSS groups (**Figure 2A**). The proportion of NK cells and monocyte-macrophages was significantly higher in the pSS group than in the control group. This finding was consistent with that of our previous study, which revealed an increased abundance of innate immune cells in LSGs (11). The general distribution patterns of different immune cell subsets in PBMCs were comparable between the control and pSS groups (**Figure 2B** **).** To determine the functions of different immune cell subsets, we compared enriched pathways between the control and pSS groups. KEGG enrichment analysis indicated that human blood monocytes and macrophages were specifically enriched in pathways associated with Th17 cell differentiation, rheumatoid arthritis, reactive oxygen species and Th1 and Th2 differentiation (**Figure 2C**). Gene Ontology (GO) analysis revealed that monocytes and macrophages were enriched in biological processes such as regulation of response to external stimulus, neutrophil chemotaxis, cell killing and myeloid leukocyte activation (**Figure 2D**). A heat map of three identified marker genes (AIF1, SERPINA1 and LST1) and nine proinflammatory factors (IL1B, IL18, NLRP3, TGFB1, TNF and NFKBIA) was generated to distinguish monocyte and macrophage subsets based on differences in their transcriptomic profiles (**Figure 2E**). The expression patterns of the marker genes and proinflammatory factors were significantly different between the control and pSS groups (**Figure 2F**). Altogether, these results suggest that monocytes and macrophages play an essential role in peripheral inflammatory responses by modulating the release of inflammatory cytokines and phagocytosis in pSS.

### Identification of M1 macrophages based on their proinflammatory functions

**Figure 3:**
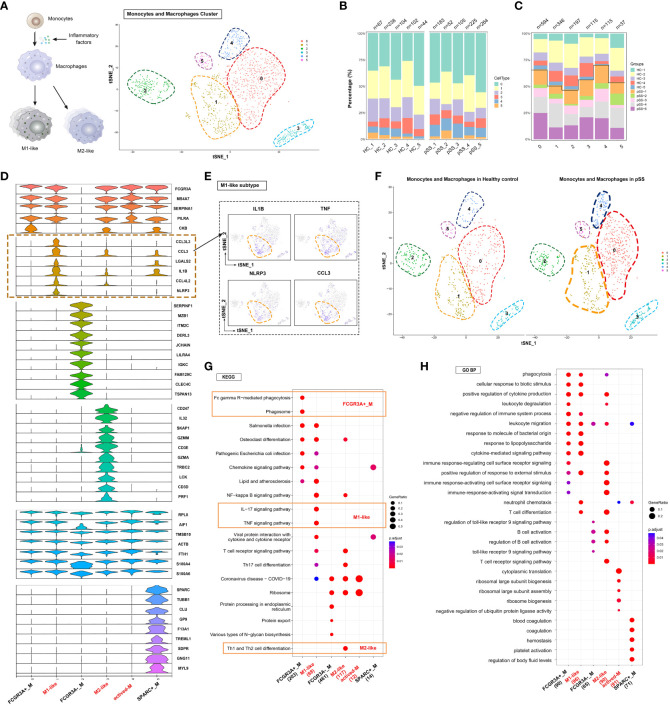
Heterogeneous subpopulations and patterns of peripheral monocyte-macrophages in patients with pSS **(A)** Classification of peripheral monocyte-macrophages into six cell subsets based on the expression of surface markers. t-SNE plots demonstrating single-cell gene expression pooled across CD68^+^ samples are shown. Clusters are labelled by cell subtypes. **(B)** Bar plot depicting the proportion of each monocyte-macrophage subset in PBMCs from healthy individuals (n = 5) and patients with pSS (n = 5). **(C)** Bar plot depicting the proportion of each monocyte-macrophage subset in each sample. The height of each bar indicates the proportion of genes in each cluster. **(D)** Violin plot demonstrating differentially expressed genes for each subset. Inflammation-related genes showed distinct expression patterns between the M1-like cluster (cluster 1) and other clusters. **(E)** t-SNE plot demonstrating the expression of representative genes (IL1B, TNF, NLRP3 and CCL3) coloured blue in the M1-like cluster (cluster 1). **(F)** t-SNE plot of monocytes and macrophages in healthy control (Left) and pSS (Right). **(G)** KEGG enrichment analysis identified the specific metabolic pathways enriched in each subset. **(H)** Gene Ontology (GO) enrichment analysis revealed biological processes associated with each subset.

The process of macrophage activation is shown in **Figure 3A**. Unsupervised hierarchical clustering was performed on 5,399 monocytes and 1,404 macrophages to examine their heterogeneity and complexity in PBMCs (**Figure 1E**). Monocytes and macrophages were clustered into six subsets in each sample (**Figure 3A**). A stacked bar plot was generated to visualise the proportion of cells in each cluster in each sample, with clusters 0 and 1 comprising the majority of macrophages (**Figure 3B**). The proportion of cells in clusters 0 and 4 was higher in the pSS group than in the control group (**Figure 3C**). Cells in cluster 0 exhibited high expression of macrophage-related genes such as FCGR3A, MS4A7 and SERPINA1. Cells in cluster 1 exhibited an inflammatory phenotype with high expression of CCL3L3, CCL3, IL1B and NLRP3, indicating the presence of M1-like macrophages (**Figures 3D, E**). Cells in cluster 2 exhibited low expression of FCGR3A and high expression of SERPINF1, MZB1, ITM2C, DERL3, JCHAIN, LILRA4 and IGKC. Cells in cluster 3 exhibited an M2-like phenotype characterised by high expression of CD247, IL32, SKAP1, GZMM, CD3E and GZMA. Cells in cluster 4 exhibited high expression of S100A4 and S100A6, with the expression of the two genes being significantly higher in the pSS group than in the control group (**Figure 3F**, **Supplementary Table 2**). Cells in cluster 5 exhibited remarkably high expression of SPARC, TUBB1, CLU, GP9, F13A1 and TREML1. KEGG and GO enrichment analyses indicated that FCGR3A^+^ cells in cluster 0 were specifically enriched in Fc gamma R-mediated phagocytosis (**Figure 3G**), whereas M1-like cells in cluster 1 were enriched in the IL-17 signalling pathway, TNF signalling pathway, neutrophil chemotaxis and positive regulation of response to external stimulus (**Figures 3G, H**). M2-like cells in cluster 3 were enriched in Th1 and Th2 cell differentiation (**Figure 3G**). Altogether, these results suggested that M1-like cells were mainly involved in immune- and inflammation-related pathways.

### Histological staining and transcriptomic profiling of macrophages isolated from LSGs

**Figure 4:**
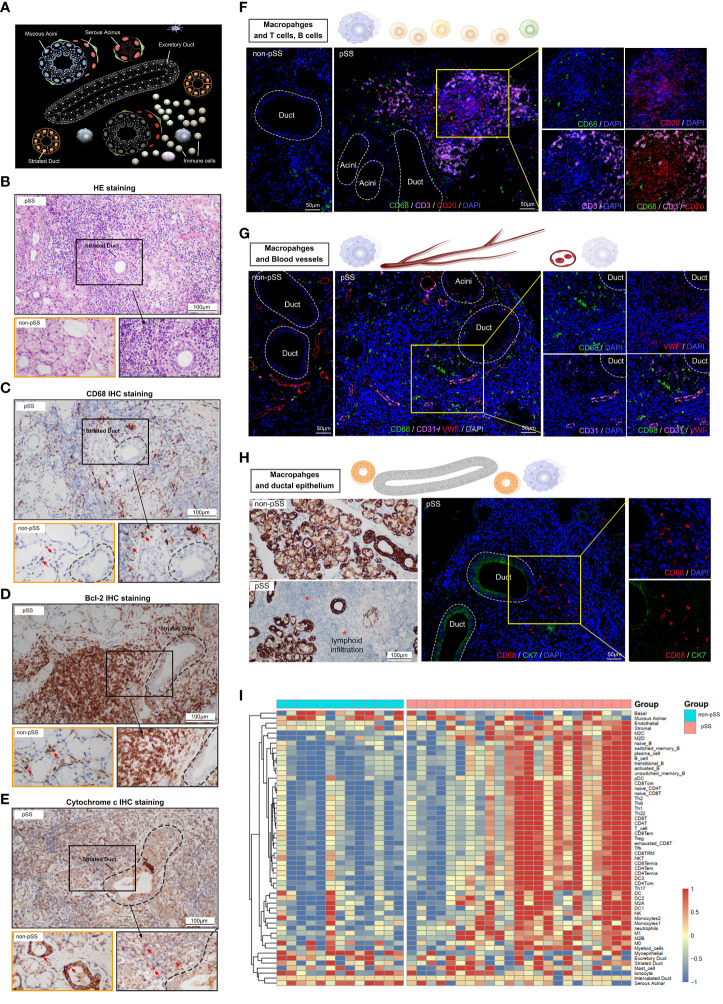
Characterisation of macrophages and the salivary gland microenvironment via histological staining and RNA-seq. **(A)** Schematic diagram depicting lymphocytic infiltration in the salivary glands. **(B)** Representative pathological images (HE staining, ×400) of tissues from patients with pSS and healthy individuals (scale bar = 100 μm). **(C–E)** Representative images of IHC staining for CD68 **(C)**, Bcl-2 **(D)** and cytochrome c **(E)** in human LSGs from patients with pSS and healthy individuals (scale bar = 100 μm). **(F)** Immunofluorescence (IF) staining of macrophages (CD68, green), T cells (CD3, magenta), B cells (CD20, red) and nuclei (DAPI, blue). Independent (right) and merged (middle) fluorescent signals are shown (scale bar = 50 μm). **(G)** Representative images of co-IF analysis of macrophages (CD68, green), blood vessels (CD31, magenta and VWF; red) and nuclei (DAPI, blue) (scale bar = 50 μm). **(H)** IHC staining for CK7 in minor salivary gland tissues (scale bar = 100 μm) (left); co-IF analysis of macrophages (CD68, red) and ductal epithelium (CK7, green) in minor salivary gland tissues (scale bar = 50 μm) (right). **(I)** Heat map demonstrating the results of ssGSEA for immune cell clusters and glandular microenvironmental factors. Each column represents an individual sample, each row represents an immune cell or factor coloured to indicate the relative abundance or expression (red, increased abundance or expression; blue, decreased abundance or expression).

Histological staining was performed to assess the tissue distribution of macrophages in LSG samples from patients with pSS and healthy individuals. As shown in **Figure 4A**, the human salivary gland tissue possesses ducts, acini and stromal components. The naïve ducts are hierarchical: Large excretory ducts branch from the main duct and into smaller striated ducts, which further branch into smaller intercalated ducts. Multiple acini are connected to a single duct (22), and immune cells are present across glandular tissues. Histological staining was performed to assess the morphological features of LSG tissues from patients with pSS and healthy individuals. A high degree of lymphocyte (**Figure 4B**) and CD68^+^ macrophage (**Figure 4C**) infiltration was observed in damaged LSG tissues from patients with pSS. Bcl-2, an anti-apoptotic factor, is upregulated in senescent cells (33) and localises diffusely in the cytoplasm. The expression of Bcl-2 was high at the lymphocyte infiltration site in damaged LSG tissues, indicating the progression of autoimmune inflammation (34). Cytochrome c is an important mitochondrial protein involved in ATP synthesis and is released into the cytosol when stimulated by apoptosis. The release of cytochrome c is tightly regulated by Bcl-2 (35). During cellular damage, cytochrome c serves as a danger-associated molecular pattern (DAMP) after being released into the extracellular space (36). Immunohistochemical (IHC) staining was performed to assess the expression of Bcl-2 and cytochrome c in LSG tissues from patients with pSS and healthy individuals. The results showed that the expression of Bcl-2 was high at lymphocyte infiltration sites in LSG tissues from patients with pSS, whereas it was weak or absent in LSG tissues from healthy individuals (**Figure 4D**). The expression of mitochondrial cytochrome c was low at lymphocyte infiltration sites in LSG tissues from patients with pSS (**Figure 4E**, **Supplementary Figure S1**). To investigate the correlation of macrophages with other immune cells, blood vessels and the ductal epithelium, immunofluorescence (IF) analysis was performed and the enrichment scores of different immune cell subsets were calculated using the ssGSEA algorithm. In the pSS group, the abundance of T and B cells was high around the ducts, with a few macrophages scattered at the infiltration site (**Figure 4F**). In addition, the abundance of macrophages was high in the perivascular (**Figure 4G**) and damaged tissues as well as the neighbouring sites (**Figure 4H**) in the pSS groups. These findings validate that macrophages are associated with immune responses and cell apoptosis. Furthermore, LSG tissues from patients with pSS and healthy individuals were subjected to high-throughput RNA sequencing, followed by bioinformatic analysis. As shown in the heat map in **Figure 4I**, the gene expression of immune cell markers was high and that of select epithelial cell markers was low in the pSS group (**Supplementary Table 3**). These results indicated that epithelial damage in pSS led to the recruitment of various immune cells, resulting in the formation of a heterogeneous glandular immune microenvironment.

### Unique gene modules associated with M1 macrophages

**Figure 5:**
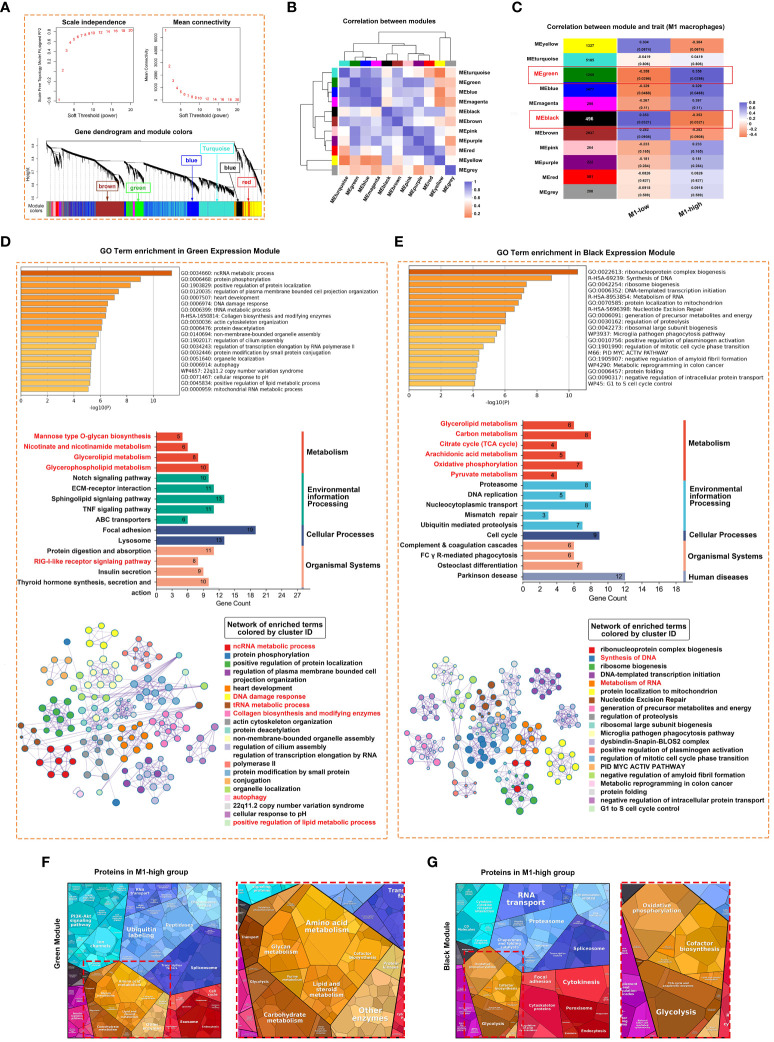
Weighted correlation network analysis (WGCNA) revealed unique gene modules associated with M1 macrophages. **(A)** Analysis of network topology for various soft thresholding powers and the mean network connectivity under different weighted coefficients (top). The hierarchical cluster tree depicts the co-expression modules identified via WGCNA (bottom). Each module corresponds to a branch, which was labelled with different colours. **(B)** Heat map demonstrating the Pearson correlation coefficients of eigengenes of co-expressed gene modules. A total of 10 modules were identified with highly correlated gene expression patterns. **(C)** The correlation between each module and M1 macrophages is indicated by orange or purple colour, respectively. The p-value for each module is shown in parentheses. **(D, E)** Horizontal bar chart representing the results of GO (upper) and KEGG (middle) analyses of genes in the green **(D)** and black **(E)** modules of M1-macrophages. The overlapping genes were further analysed using Metascape. **(F, G)** Heat map demonstrating the differential functional categories of genes in green **(F)** and black **(G)** modules as illustrated by Proteomaps based on RNA-seq data. Each KEGG pathway is represented by a polygon-shaped tile; pathways belonging to the same class share similar colours and are arranged adjacently to form larger regions.

Weighted gene co-expression network analysis (WGCNA) was performed to identify gene co-expression networks associated with M1 macrophages. The optimal soft thresholding power was selected to generate block-wise modules (**Figure 5A**). A total of 10 RNA expression modules were identified and assigned a unique colour-coded identifier (grey indicated genes that did not belong to any known module), with each identifier representing a characteristic expression pattern. Pearson correlation coefficients were computed to examine the relationship among the gene modules (**Figure 5B**). The green module was positively correlated with the M1 macrophage phenotype, whereas the black module was negatively correlated with the M1 macrophage phenotype (**Figure 5C**). The modules and their hub genes were further analysed to gain insights into the pathogenesis of pSS. Consistent with the aforementioned results, genes in the green module were primarily enriched in ncRNA metabolic process, protein phosphorylation, DNA damage response, RIG-I-like receptor signalling pathway, collagen biosynthesis, autophagy and positive regulation of lipid metabolic process (**Figure 5D**). Genes in the black module were primarily enriched in ribonucleoprotein complex biogenesis, synthesis of DNA and metabolism of RNA (**Figure 5E**). To examine specifically for signatures of metabolic pathways, we analysed enrichment of metabolic networks. M1 macrophages were positively correlated with multiple metabolic pathways, including glycerophospholipid metabolism, glycerolipid metabolism, amino acid metabolism, glycan metabolism and carbohydrate metabolism (**Figures 5D, F**). In addition, KEGG pathway analysis revealed that M1-low group was more markedly enriched in the citrate cycle (TCA cycle), oxidative phosphorylation, pyruvate metabolism and glycolysis (**Figures 5E, G**).

### M1-like macrophages are associated with the reprogramming of inflammatory, mitochondrial and metabolic pathways as part of innate immune responses

**Figure 6:**
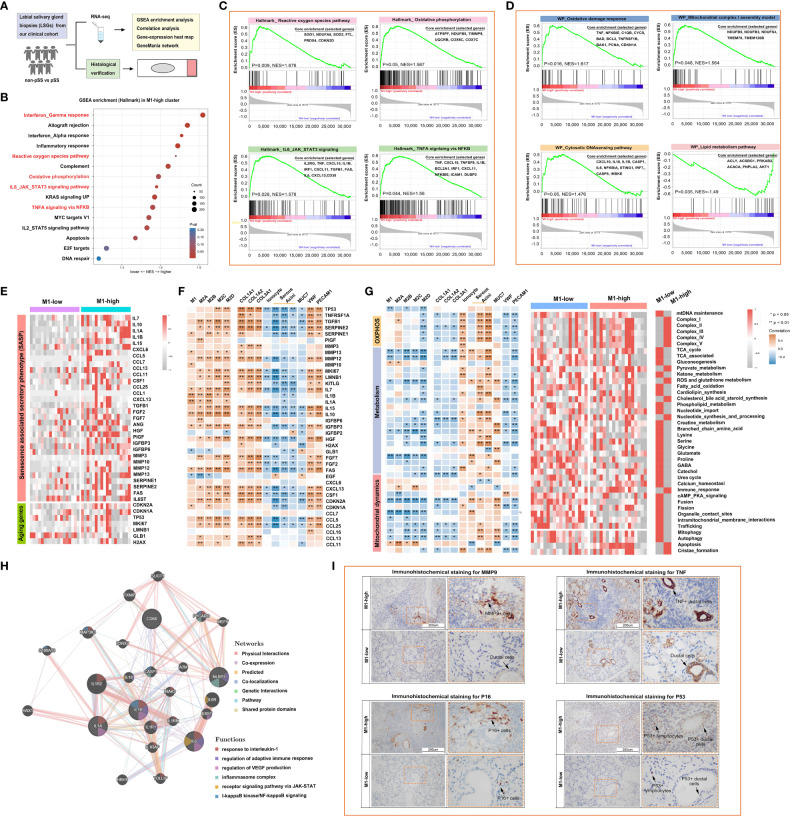
M1-like macrophages are associated with the reprogramming of inflammatory, mitochondrial and metabolic pathways as part of the innate immune response. **(A)** Schematic outline of clinical study design. A total of 36 participants, including 12 healthy individuals and 24 patients with pSS, were enrolled. **(B)** Bubble plot demonstrating the results of gene set enrichment analysis (GSEA) for major hallmark pathways enriched in the high- and low-M1-macrophage-infiltration groups. Normalised enrichment scores (NESs) are shown. Colour coding corresponds to adjusted p-values. **(C, D)** Heat map demonstrating the results of GSEA for genes involved in the regulation of inflammatory responses **(C)** and mitochondrial metabolic pathways **(D)**. **(E)** Heat map demonstrating the expression of inflammation- and ageing-related genes in the high- and low-M1-macrophage-infiltration groups based on RNA-seq data. **(F)** Correlation of macrophages with inflammation- and ageing-related genes. **(G)** Heat map demonstrating the correlation of mitochondrial metabolic pathways with the glandular microenvironment (left) and the expression of metabolic pathway-related genes (right) in the high- and low-M1-macrophage-infiltration groups based on RNA-seq data. **(H)** Co-expression network depicting potential relationships between macrophages and complex inflammatory pathways as generated using GeneMANIA. **(I)** Representative images of IHC staining for MMP9, TNF, P16 and P53 in human LSGs in the high- and low-M1-macrophage-infiltration groups (scale bar = 200 μm).

A flowchart demonstrating the protocol of RNA sequencing and histological staining in the pSS and control groups is shown in **Figure 6A**. To determine the biological pathways through which M1 macrophages contribute to the development of pSS, GSEA was performed based on RNA-seq data. Samples were divided into high- and low-M1-macrophage-infiltration groups. The results of GSEA indicated that Hallmark genes were enriched in IFN-γ response, reactive oxygen species pathway, oxidative phosphorylation, IL-6–JAK–STAT3 signalling pathway and TNFA signalling via NFKB in the low-M1-macrophage-infiltration group (**Figures 6B, C**). Consistently, WIKI-pathway genes were primarily enriched in pathways related to inflammation, tissue damage and metabolism, including oxidative damage response, mitochondrial complex I assembly and cytosolic DNA-sensing pathway, in the high-M1-macrophage-infiltration group, whereas the genes were enriched in the lipid metabolism pathway in the low-M1-macrophage-infiltration group (**Figure 6D**). Because the results of GSEA revealed that M1 macrophages were primarily involved in proinflammatory pathways, we analysed the relationship among macrophages, ageing and metabolism in pSS. As anticipated, the relative expression of senescence-associated secretory phenotype (SASP) and ageing-related genes was upregulated in the high-M1-macrophage-infiltration group (**Figures 6E, F**). Subsequently, the ssGSEA algorithm was used to evaluate the enrichment scores of mitochondrial metabolic pathways identified using the Mitocarta 3.0 database. As shown in the heat map in **Figure 6G**, metabolic pathways such as the TCA cycle, gluconeogenesis, amino acid metabolism and mitochondrial dynamics were remarkably downregulated in the high-M1-macrophage-infiltration group. These results suggest that reprogramming of mitochondrial metabolic pathways plays an important role in pSS. Additionally, the co-expression network constructed using GeneMANIA depicted several potential associations between macrophages and inflammatory signalling pathways (**Figure 6H**). IHC staining showed that the inflammatory factors MMP and TNF were upregulated in the high-M1-macrophage-infiltration group, which was consistent with the results of RNA-seq (**Figure 6I**, **Supplementary Figure S2**). DNA damage is a major factor contributing to cell ageing. Upon DNA damage, the p53 and p16 proteins arrest the cell cycle and activate DNA repair proteins. The expression of p53 and p16 was high in the ductal cells of the high-M1-macrophage-infiltration group, whereas it was minimal in the control group (**Figure 6I**). These results indicate that tissue-resident M1 macrophages enhance cytokine production and play a crucial role in inflammation, ageing and metabolism.

### Macrophage responses are strongly associated with the glandular immune microenvironment

**Figure 7:**
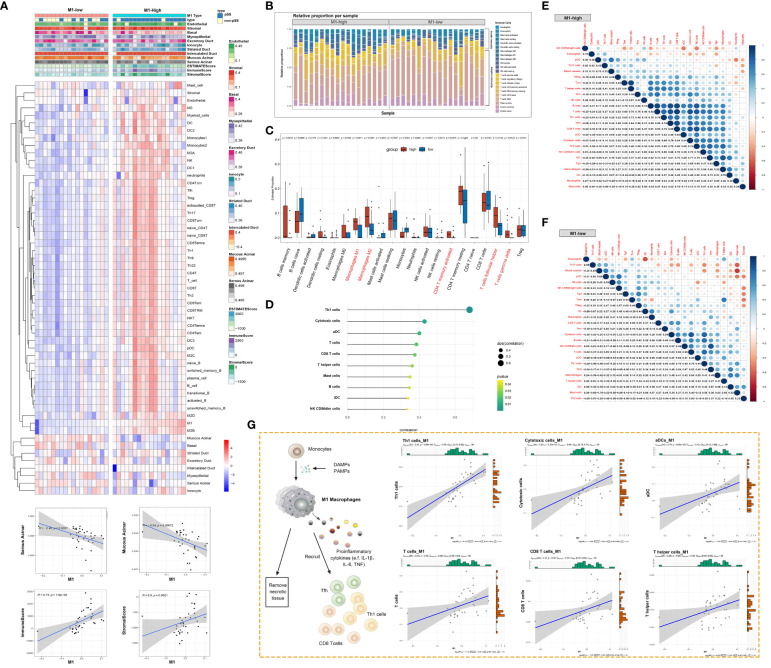
Macrophage responses are strongly associated with the glandular immune microenvironment. **(A)** Heat map demonstrating the immune cell infiltration landscape in the high- and low-M1-macrophage-infiltration groups based on the results of the ssGSEA and ESTIMATE algorithms and unsupervised hierarchical clustering (top). Scatter plot depicts the correlation between M1 macrophages and genes in the glandular microenvironment (bottom). **(B)** Stacked bar plot demonstrating the proportion of tissue-infiltrating innate and adaptive immune cells assessed using the CIBERSORT algorithm. **(C)** Violin plot depicting the results of the CIBERSORT algorithm in two clusters; p-values of <0.05 (red) were considered statistically significant. **(D)** Lollipop chart demonstrating the correlation between M1 macrophages and other immune cells. **(E, F)** Heat map demonstrating the correlation between the infiltration levels of different innate and adaptive immune cells in the high-M1-macrophage infiltration **(E)** and low-M1-macrophage infiltration **(F)** groups. Pearson correlation coefficients are shown (blue, positive correlation; orange, negative correlation). **(G)** Schematic diagram representing the differentiation of monocytes into M1 macrophages, facilitating the adaptive immune response (left). Scatter plot represents the relationship between M1 macrophages and six types of immune cells.

The immune response and immune microenvironment play an important role in the initiation, progression and treatment response of diseases. The ssGSEA algorithm was used to quantify glandular tissue-infiltrating immune cells, and unsupervised hierarchical clustering was performed on 45 immune cells and 10 glandular components based on the RNA-seq data of LSG tissues from patients with pSS and healthy individuals. The abundance of most immune cells, including T cells, B cells and DCs, was high in the high-M1-macrophage-infiltration group (**Figure 7A**, **Supplementary Table 4**). Adaptive immune cells account for the majority of tissue-resident immune cells in salivary glands (**Figure 7B**). However, the glandular microenvironment developed within lymphoid infiltration foci is thought to have dysregulated interactions between innate and adaptive immune system components, thereby affecting the protective function of resident immune cells. The abundance of activated memory CD4^+^ T cells, follicular helper T (Tfh) cells and gamma-delta T cells was higher in the high-M1-macrophage-infiltration group than in the low-M1-macrophage-infiltration group (**Figure 7C**). M1 macrophages were positively correlated with several immune cells, including Th1 cells, cytotoxic cells, aDCs, CD8 T cells, T helper cells, mast cells, B cells, iDCs and CD56^dim^ NK cells (P < 0.05) (**Figure 7D**). The degree of immune cell infiltration and the relationship among immune cells were more significant in the high-M1-macrophage-infiltration group (**Figures 7E, F**). These results suggest that tissue-resident M1 macrophages may be derived, at least partly, from ‘M1-like’ monocytes/macrophages under the influence of proinflammatory cytokines (such as DAMPs and pathogen-associated molecular patterns [PAMPs]). Upon activation, M1 macrophages clear necrotic tissues and release immune mediators that actively interact with other immune cells (**Figure 7G**).

## Discussion

Over-activation of innate immune cells is associated with the pathogenesis of pSS (37), and epigenetic and metabolic reprogramming essentially contribute to the hyperresponsiveness of the immune system (38, 39). Here, we selected macrophages as the focus of the immune microenvironment and examined their infiltration patterns, polarisation status and metabolic reprogramming in pSS. The composition of immune cells, especially monocyte-macrophage subpopulations, in PBMCs from healthy individuals and patients with pSS was assessed using single-cell transcriptomic data from a GEO dataset. Monocyte-macrophages, especially M1-like macrophages, were found to be involved in multiple inflammation-related pathways. Histological staining revealed the distribution patterns of macrophages in clinical LSG tissues from healthy individuals and patients with pSS. Given the key role of M1 macrophages in inflammation in pSS, the samples were further divided into high- and low-M1-macrophage-infiltration groups. Metabolic pathways and infiltration patterns were substantially different between the two groups, indicating that macrophages undergo metabolic reprogramming in pSS. Therefore, M1 macrophages may serve as a promising therapeutic target for pSS.

Autoimmune disease results from immune responses evoked by self-antigens that lead to the disruption of immune homeostasis (40), accompanied by the immune tolerance breakdown in body tissue, which means loss of the immune system’s ability to prevent itself from targeting its own molecules, cells, or tissues (41), and disorder of immune balance (42). When lymphocytes fail to recognise non-self-antigens correctly, they may attack various organs and tissues of the body, leading to their degeneration, damage and loss of function (43). Immune cell infiltration into autoimmune target organs occurs in two ways: recruitment of peripheral blood progenitor cells and proliferation of resident precursor cells (44). Many previous studies have demonstrated that PBMCs play an important role in the pathogenesis of autoimmune diseases (45–47). In this study, peripheral immune population differences distinguishing pSS from healthy control were primarily found in innate immune cells. The abundance of NK cells and monocyte-macrophages was higher in the pSS group than in the control group. Furthermore, functional enrichment analysis revealed that monocyte-macrophages were primarily involved in inflammation-related signalling pathways, including myeloid leukocyte activation, Epstein–Barr virus infection, cell killing and NK cell-mediated immunity and cytotoxicity. NK cells, a type of innate immune cell, co-localise with macrophages in the splenic red pulp and peripheral lymph nodes, suggesting their interaction with macrophages (48). NK cells can negatively regulate hyperactivated macrophages. In addition, NK cells and macrophages collectively participate in the production of IFN-γ, promoting protective immunity during the early stage of infection (49). Haematopoietically derived cells such as macrophages play diverse and crucial roles in the pathogenesis of autoimmune and inflammatory diseases. They can not only clear pathogens and alleviate inflammation by removing necrotic cell debris but also enhance inflammation by secreting proinflammatory mediators and transmitting proinflammatory signals in pathological conditions (50). Given that macrophages are major participants in host defence and inflammatory responses, dysregulation of macrophage differentiation and function may lead to multiple human diseases, including cancer and autoimmune disorders (51).

Activated macrophages can be divided into two groups: classically activated macrophages (M1 macrophages), which induce proinflammatory responses, and alternatively activated macrophages (M2 macrophages) which exert anti-inflammatory effects and promote tissue repair (52, 53). In this study, we identified six macrophage subsets in PBMCs from healthy individuals and patients with pSS based on scRNA-seq data. In particular, one of the subsets specifically expressed many important inflammatory factors, including IL1B, TNF, CCL3 and NLRP3 inflammasome. Because the proinflammatory functions of this subset were similar to those of M1 macrophages, we annotated it as M1‐like macrophages. The expression of multiple inflammation-related genes was upregulated in the M1-like subset, whereas genes in the M2-like subset were mainly involved in T-cell differentiation, B-cell activation and immune response-activation signal transduction. As important mediators of inflammatory responses, proinflammatory cytokines trigger an inflammatory cascade in autoimmune diseases (49). They are critically involved in regulating the immune response and recruiting immune cells. NLRP3 is a pattern recognition receptor (PRR) that triggers IL-1β secretion when activated by various exogenous and endogenous stimuli (54, 55). Circulating macrophages alter their phenotype and effector functions in response to external stimuli. In the presence of cytokines, such as IFN-γ, or lipopolysaccharides (LPSs), M1 macrophages can upregulate the pentose phosphate pathway to increase ROS levels (56), consequently killing pathogens and clearing necrotic tissue. In addition, macrophages can produce inflammatory cytokines such as IL-1, IL-6, IL-23 and TNF (53). Unlike M1 macrophages, M2 macrophages produce anti-inflammatory cytokines and increase the expression of arginase-1 (Arg1) when stimulated by Th2-type cytokines (IL-4, IL-10 and IL-13), thereby attenuating inflammatory responses and promoting wound healing (57–59). Although the mechanisms through which activated macrophages differentiate into M1 and M2 phenotypes to regulate immune responses remain unclear, several preclinical studies focusing on normalising the M2-to-M1 ratio in autoimmune diseases are underway (60).

In this study, we assessed the localisation and expression of cytochrome c in healthy and damaged salivary gland tissues. Mitochondrial damage in pSS downregulated the expression of cytochrome c in ductal epithelial cells and promoted its release into the periductal area, resulting in lymphocyte recruitment. Inflammatory infiltration initially resembled small clusters around the ducts but subsequently expanded to form structures resembling ectopic germinal centres (GCs) in LSG tissues from patients with pSS (61). Characterised by chronic inflammation, ageing and hypoxia, the immune microenvironment of salivary glands in pSS was dominated by CD4^+^ T cells, with the abundance of CD8^+^ T cells, B cells and plasma cells being relatively low. Complex interactions between infiltrating immune cells and tissue-resident cells facilitate disease progression in a location- and time-dependent manner (61, 62). Human monocytes constitute approximately 10% of peripheral blood leukocytes (63). During inflammation, peripheral blood monocytes migrate to tissues and differentiate into resident macrophages (64). These macrophages are polarised in response to microenvironmental signals to eliminate apoptotic cells and cellular debris. This phenomenon is considered to be involved in the development of autoimmune disorders (65). In this study, macrophages were found to be widely distributed in injured ducts and acini and co-localised with other important immune cells (including T cells and B cells) in the glandular immune microenvironment. Recent studies have shown that the abundance of M1 macrophages and expression of related inflammatory factors are high in the early stage of pSS, whereas M2 macrophages appear in the later stage of the disease and mediate chronic inflammation leading to irreversible salivary gland fibrosis (66, 67). In addition, macrophages participate in CD4^+^ T-cell activation (66, 68) and play an indispensable role in the development and maturation of B cells. Deletion of macrophages results in poor Tfh cell activation (69). In this study, the abundance of DCs was positively associated with the abundance of M1 macrophages. DCs play an important role in initiating the disruption of immune homeostasis and the formation of an autoimmune environment. They participate in disease development mainly by producing type I interferons and presenting antigens (70) and have been shown to infiltrate the salivary glands in pSS (71). Macrophages and DCs are closely related cell populations generated from a common myeloid progenitor in the bone marrow (58). Several proinflammatory cytokines produced by DCs and macrophages in target tissues in pSS are closely associated with the development of inflammatory lesions. IL-18 expression in macrophages has been associated with the abundance of both infiltrating DC cells and macrophages (72). Macrophages coordinate immune responses through complex bidirectional interplay with glandular cells and other immune cells.

In this study, macrophages were found to be distributed near many micro-vessels in LSG tissues from patients with pSS. This finding indicates that inflammatory macrophages may interact with damaged vessels in pSS. Upregulated serum CXCL4 in patients with pSS inhibits endothelial cell proliferation, which is closely related to microvascular injury (73). Macrophages produce vascular endothelial growth factor-A (VEGF-A), stimulating angiogenesis during chronic inflammation (74). On the contrary, vascular endothelial cells recruit circulating monocytes to the site of vascular injury, further enhancing the immune response (75). Furthermore, phagocytosis of necrotic tissue by macrophages was evidenced by their aggregation at the sites of ductal damage. Altogether, these findings reveal that the immune microenvironment of salivary glands in pSS is characterised by the increased abundance of various immune cells and impairment of glandular function. Moreover, pSS progression is influenced by complex interactions between macrophages and other components in the microenvironment.

Metabolic pathways associated with immune cells were remarkably different between the high- and low-M1-macrophage-infiltation groups, which is consistent with the results of a previous study (76). The pathways enriched in the module exhibiting the strongest positive correlation with M1-high group revealed pathways of cells in pSS salivary gland microenvironment with high expression of M1-like macrophage. Unlike M2 macrophages, M1 macrophages can cause DNA damage by producing excessive ROS (77). Lipid metabolism plays a vital role in regulating the function of macrophages involved in energy metabolism and signal transduction in activation process (78). Metabolic reprogramming of macrophages significantly contributes to the development of pSS (79). Under homeostatic conditions, macrophages produce ATP primarily through the TCA cycle and oxidative phosphorylation (OXPHOS). The hallmark of metabolic reprogramming in activated macrophages is the reduced utilisation of the TCA cycle and OXPHOS, with increased amino acid metabolism. Alterations in macrophage metabolism may be related to their proinflammatory function. For example, the transition from OXPHOS to glycolysis results in increased IL-1β production in macrophages (80). Increased cofactor biosynthesis associated with M1-macrophage activation may be related to the production of NADPH for NO and ROS production or nucleotides for RNA synthesis (81). The expression of senescence-related genes was upregulated in the high-M1-macrophage-infiltration group. In addition, the senescence markers p16 and p53 were expressed in lymphocytes, indicating immunosenescence in the inflammatory microenvironment. Immunosenescence can manifest as impaired immune function and immune cell senescence, with the increased expression of SASP in NK cells, T cells and macrophages (82). The expression of inflammation-related genes was positively associated with the abundance of macrophages and negatively associated with glandular function in pSS. However, the activity of mitochondria-related pathways exhibited the opposite trend, indicating the role of macrophages in the immune microenvironment of salivary glands in pSS. Altogether, targeting M1-like macrophages is a promising strategy for effective treatment of autoimmune diseases such as pSS.

This study has certain limitations that should be acknowledged. Owing to the limited sample size, the results of this study should be validated in future studies with large sample sizes. In addition, the identification of differential pathways in this study was based on the entire salivary gland microenvironment. Therefore, the function of each cell should be further verified via single-cell analysis and future studies should identify effective drugs targeting M1-like macrophages for the treatment of pSS.

## Conclusion

This study suggests that macrophages are among the most abundant innate immune cells in the PBMCs of patients with pSS. In particular, M1-like macrophages play a key role in inflammation in pSS. A bidirectional relationship exists between macrophage polarisation and the inflammatory microenvironment, and macrophages undergo metabolic reprogramming in the inflammatory microenvironment. Therefore, M1 macrophage-targeted therapy may represent an effective strategy for treating pSS.

## Data availability statement

The datasets presented in this study can be found in online repositories. The names of the repository/repositories and accession number(s) can be found in the article/**Supplementary Material**. The data presented in the study are deposited in the Bio-Med Big Data Center repository, accession number OED843206.

## Ethics statement

The studies involving humans were approved by the ethics committee of Ruijin Hospital, Shanghai Jiao Tong University School of Medicine and Chinese Clinical Trial Registry (ChiCTR2000039820). The studies were conducted in accordance with the local legislation and institutional requirements. The participants provided their written informed consent to participate in this study.

## Author contributions

YZ: Writing – original draft. YY: Writing – original draft, Formal Analysis, Methodology, Software, Visualization. JZ: Writing – original draft, Methodology. LL: Writing – original draft, Methodology, Validation. DL: Writing – original draft. JH: Writing – original draft. YG: Writing – original draft. LW: Writing – original draft, Writing – review & editing. NL: Writing – original draft, Writing – review & editing. LJ: Writing – original draft, Writing – review & editing, Conceptualization.

## References

[1] Nocturne G Mariette X. B cells in the pathogenesis of primary sjogren syndrome. Nat Rev Rheumatol (2018) 14(3):133–45. doi: 10.1038/nrrheum.2018.1 29416129

[2] Qin B Wang J Yang Z Yang M Ma N Huang F. Epidemiology of primary sjögren’s syndrome: A systematic review and meta-analysis. Ann rheumatic Dis (2015) 74(11):1983–9. doi: 10.1136/annrheumdis-2014-205375 24938285

[3] Witas R Shen Y Nguyen CQ. Bone marrow-derived macrophages from a murine model of sjogren’s syndrome demonstrate an aberrant, inflammatory response to apoptotic cells. Sci Rep (2022) 12(1):8593. doi: 10.1038/s41598-022-12608-4 35597820 PMC9124194

[4] Kassan SS Moutsopoulos HM. Clinical manifestations and early diagnosis of sjögren syndrome. Arch Internal Med (2004) 164(12):1275–84. doi: 10.1001/archinte.164.12.1275 15226160

[5] Delaleu N Jonsson R Koller MM. Sjögren’s syndrome. Eur J Oral Sci (2005) 113(2):101–13. doi: 10.1111/j.1600-0722.2004.00183.x 15819815

[6] Brito-Zeron P Baldini C Bootsma H Bowman SJ Jonsson R Mariette X. Sjogren syndrome. Nat Rev Dis Primers (2016) 2:16047. doi: 10.1038/nrdp.2016.47 27383445

[7] Tzioufas AG Kapsogeorgou EK Moutsopoulos HM. Pathogenesis of sjogren’s syndrome: what we know and what we should learn. J Autoimmun (2012) 39(1-2):4–8. doi: 10.1016/j.jaut.2012.01.002 22326205

[8] Manoussakis MN Kapsogeorgou EK. The role of intrinsic epithelial activation in the pathogenesis of sjögren’s syndrome. J Autoimmun (2010) 35(3):219–24. doi: 10.1016/j.jaut.2010.06.011 20685080

[9] Okuma A Hoshino K Ohba T Fukushi S Aiba S Akira S. Enhanced apoptosis by disruption of the stat3-ikappab-zeta signaling pathway in epithelial cells induces sjogren’s syndrome-like autoimmune disease. Immunity (2013) 38(3):450–60. doi: 10.1016/j.immuni.2012.11.016 23453632

[10] Verstappen GM Pringle S Bootsma H Kroese FGM. Epithelial-immune cell interplay in primary sjögren syndrome salivary gland pathogenesis. Nat Rev Rheumatol (2021) 17(6):333–48. doi: 10.1038/s41584-021-00605-2 PMC8081003 33911236

[11] Luo D Li L Wu Y Yang Y Ye Y Hu J. Mitochondria-related genes and metabolic profiles of innate and adaptive immune cells in primary sjögren’s syndrome. Front Immunol (2023) 14:1156774. doi: 10.3389/fimmu.2023.1156774 37497211 PMC10366690

[12] Nocturne G Mariette X. Advances in understanding the pathogenesis of primary sjögren’s syndrome. Nat Rev Rheumatol (2013) 9(9):544–56. doi: 10.1038/nrrheum.2013.110 23857130

[13] Ginhoux F Schultze JL Murray PJ Ochando J Biswas SK. New insights into the multidimensional concept of macrophage ontogeny, activation and function. Nat Immunol (2016) 17(1):34–40. doi: 10.1038/ni.3324 26681460

[14] Metschnikoff E. Lecture on phagocytosis and immunity. Br Med J (1891) 1(1570):213–7. doi: 10.1136/bmj.1.1570.213 PMC2197023 20753232

[15] Christofides A Strauss L Yeo A Cao C Charest A Boussiotis VA. The complex role of tumor-infiltrating macrophages. Nat Immunol (2022) 23(8):1148–56. doi: 10.1038/s41590-022-01267-2 PMC10754321 35879449

[16] Geissmann F Manz MG Jung S Sieweke MH Merad M Ley K. Development of monocytes, macrophages, and dendritic cells. Sci (New York NY) (2010) 327(5966):656–61. doi: 10.1126/science.1178331 PMC2887389 20133564

[17] Wculek SK Heras-Murillo I Mastrangelo A Mananes D Galan M Miguel V. Oxidative phosphorylation selectively orchestrates tissue macrophage homeostasis. Immunity (2023) 56(3):516–30 e9. doi: 10.1016/j.immuni.2023.01.011 36738738

[18] Stolp B Thelen F Ficht X Altenburger LM Ruef N Inavalli V. Salivary gland macrophages and tissue-resident cd8(+) T cells cooperate for homeostatic organ surveillance. Sci Immunol (2020) 5(46):eaaz4371–00. doi: 10.1126/sciimmunol.aaz4371 32245888

[19] Ushio A Arakaki R Otsuka K Yamada A Tsunematsu T Kudo Y. Ccl22-producing resident macrophages enhance T cell response in sjogren’s syndrome. Front Immunol (2018) 9:2594. doi: 10.3389/fimmu.2018.02594 30467506 PMC6236111

[20] Willeke P Gaubitz M Schotte H Maaser C Domschke W Schluter B. Increased serum levels of macrophage migration inhibitory factor in patients with primary sjogren’s syndrome. Arthritis Res Ther (2007) 9(2):R43. doi: 10.1186/ar2182 17470266 PMC1906791

[21] Shiboski CH Shiboski SC Seror R Criswell LA Labetoulle M Lietman TM. 2016 american college of rheumatology/european league against rheumatism classification criteria for primary sjogren’s syndrome: A consensus and data-driven methodology involving three international patient cohorts. Arthritis Rheumatol (Hoboken NJ) (2017) 69(1):35–45. doi: 10.1002/art.39859 PMC5650478 27785888

[22] Li N Ye Y Wu Y Li L Hu J Luo D. Alterations in histology of the aging salivary gland and correlation with the glandular inflammatory microenvironment. iScience (2023) 26(5):106571. doi: 10.1016/j.isci.2023.106571 37124415 PMC10131127

[23] Becht E McInnes L Healy J Dutertre CA Kwok IWH Ng LG. Dimensionality reduction for visualizing single-cell data using umap. Nat Biotechnol (2018) 37:38–44. doi: 10.1038/nbt.4314 30531897

[24] Aran D Looney AP Liu L Wu E Fong V Hsu A. Reference-based analysis of lung single-cell sequencing reveals a transitional profibrotic macrophage. Nat Immunol (2019) 20(2):163–72. doi: 10.1038/s41590-018-0276-y PMC6340744 30643263

[25] Langfelder P Horvath S. Wgcna: an R package for weighted correlation network analysis. BMC Bioinf (2008) 9:559. doi: 10.1186/1471-2105-9-559 PMC2631488 19114008

[26] Shannon P Markiel A Ozier O Baliga NS Wang JT Ramage D. Cytoscape: A software environment for integrated models of biomolecular interaction networks. Genome Res (2003) 13(11):2498–504. doi: 10.1101/gr.1239303 PMC403769 14597658

[27] Zhou Y Zhou B Pache L Chang M Khodabakhshi AH Tanaseichuk O. Metascape provides a biologist-oriented resource for the analysis of systems-level datasets. Nat Commun (2019) 10(1):1523. doi: 10.1038/s41467-019-09234-6 30944313 PMC6447622

[28] Liebermeister W Noor E Flamholz A Davidi D Bernhardt J Milo R. Visual account of protein investment in cellular functions. Proc Natl Acad Sci U.S.A. (2014) 111(23):8488–93. doi: 10.1073/pnas.1314810111 PMC4060655 24889604

[29] Rath S Sharma R Gupta R Ast T Chan C Durham TJ. Mitocarta3.0: an updated mitochondrial proteome now with sub-organelle localization and pathway annotations. Nucleic Acids Res (2021) 49(D1):D1541–d7. doi: 10.1093/nar/gkaa1011 PMC7778944 33174596

[30] Barbie DA Tamayo P Boehm JS Kim SY Moody SE Dunn IF. Systematic rna interference reveals that oncogenic kras-driven cancers require tbk1. Nature (2009) 462(7269):108–12. doi: 10.1038/nature08460 PMC2783335 19847166

[31] Zhang X Lan Y Xu J Quan F Zhao E Deng C. Cellmarker: A manually curated resource of cell markers in human and mouse. Nucleic Acids Res (2019) 47(D1):D721–D8. doi: 10.1093/nar/gky900 PMC6323899 30289549

[32] Yoshihara K Shahmoradgoli M Martínez E Vegesna R Kim H Torres-Garcia W. Inferring tumour purity and stromal and immune cell admixture from expression data. Nat Commun (2013) 4:2612. doi: 10.1038/ncomms3612 24113773 PMC3826632

[33] Salminen A Kaarniranta K Kauppinen A. Beclin 1 interactome controls the crosstalk between apoptosis, autophagy and inflammasome activation: impact on the aging process. Ageing Res Rev (2013) 12(2):520–34. doi: 10.1016/j.arr.2012.11.004 23220384

[34] Singh R Letai A Sarosiek K. Regulation of apoptosis in health and disease: the balancing act of bcl-2 family proteins. Nat Rev Mol Cell Biol (2019) 20(3):175–93. doi: 10.1038/s41580-018-0089-8 PMC7325303 30655609

[35] Ow YP Green DR Hao Z Mak TW. Cytochrome C: functions beyond respiration. Nat Rev Mol Cell Biol (2008) 9(7):532–42. doi: 10.1038/nrm2434 18568041

[36] Eleftheriadis T Pissas G Liakopoulos V Stefanidis I. Cytochrome C as a potentially clinical useful marker of mitochondrial and cellular damage. Front Immunol (2016) 7:279. doi: 10.3389/fimmu.2016.00279 27489552 PMC4951490

[37] Huijser E van Helden-Meeuwsen CG Grashof DGB Tarn JR Brkic Z Huisman JMA. Trained immunity in primary sjögren’s syndrome: linking type I interferons to a pro-atherogenic phenotype. Front Immunol (2022) 13:840751. doi: 10.3389/fimmu.2022.840751 35860283 PMC9289449

[38] Cheng SC Quintin J Cramer RA Shepardson KM Saeed S Kumar V. Mtor- and hif-1α-mediated aerobic glycolysis as metabolic basis for trained immunity. Sci (New York NY) (2014) 345(6204):1250684. doi: 10.1126/science.1250684 PMC4226238 25258083

[39] Quintin J Saeed S Martens JHA Giamarellos-Bourboulis EJ Ifrim DC Logie C. Candida albicans infection affords protection against reinfection via functional reprogramming of monocytes. Cell Host Microbe (2012) 12(2):223–32. doi: 10.1016/j.chom.2012.06.006 PMC3864037 22901542

[40] Zeng L Yang T Yang K Yu G Li J Xiang W. Curcumin and curcuma longa extract in the treatment of 10 types of autoimmune diseases: A systematic review and meta-analysis of 31 randomized controlled trials. Front Immunol (2022) 13:896476. doi: 10.3389/fimmu.2022.896476 35979355 PMC9376628

[41] Wang L Wang FS Gershwin ME. Human autoimmune diseases: A comprehensive update. J Internal Med (2015) 278(4):369–95. doi: 10.1111/joim.12395 26212387

[42] Soyer OU Akdis M Ring J Behrendt H Crameri R Lauener R. Mechanisms of peripheral tolerance to allergens. Allergy (2013) 68(2):161–70. doi: 10.1111/all.12085 23253293

[43] Gonzalez-Martin A Adams BD Lai M Shepherd J Salvador-Bernaldez M Salvador JM. The microrna mir-148a functions as a critical regulator of B cell tolerance and autoimmunity. Nat Immunol (2016) 17(4):433–40. doi: 10.1038/ni.3385 PMC4803625 26901150

[44] Ajami B Bennett JL Krieger C McNagny KM Rossi FM. Infiltrating monocytes trigger eae progression, but do not contribute to the resident microglia pool. Nat Neurosci (2011) 14(9):1142–9. doi: 10.1038/nn.2887 21804537

[45] Jung SM Lee J Baek SY Lee JH Lee J Park KS. The interleukin 33/st2 axis in patients with primary sjögren syndrome: expression in serum and salivary glands, and the clinical association. J Rheumatol (2015) 42(2):264–71. doi: 10.3899/jrheum.140234 25512474

[46] Wilkinson MGL Moulding D McDonnell TCR Orford M Wincup C Ting JYJ. Role of cd14+ Monocyte-derived oxidised mitochondrial DNA in the inflammatory interferon type 1 signature in juvenile dermatomyositis. Ann rheumatic Dis (2023) 82(5):658–69. doi: 10.1136/ard-2022-223469 PMC10176342 36564154

[47] Li Y Wang Z Han F Zhang M Yang T Chen M. Single-cell transcriptome analysis profiles cellular and molecular alterations in submandibular gland and blood in igg4-related disease. Ann rheumatic Dis (2023) 82:1348–58. doi: 10.1136/ard-2023-224363 37474274

[48] Grégoire C Chasson L Luci C Tomasello E Geissmann F Vivier E. The trafficking of natural killer cells. Immunol Rev (2007) 220(1):169–82. doi: 10.1111/j.1600-065X.2007.00563.x PMC7165697 17979846

[49] van Dommelen SL Sumaria N Schreiber RD Scalzo AA Smyth MJ Degli-Esposti MA. Perforin and granzymes have distinct roles in defensive immunity and immunopathology. Immunity (2006) 25(5):835–48. doi: 10.1016/j.immuni.2006.09.010 17088087

[50] Boada-Romero E Martinez J Heckmann BL Green DR. The clearance of dead cells by efferocytosis. Nat Rev Mol Cell Biol (2020) 21(7):398–414. doi: 10.1038/s41580-020-0232-1 32251387 PMC7392086

[51] Mantovani A Allavena P Sica A Balkwill F. Cancer-related inflammation. Nature (2008) 454(7203):436–44. doi: 10.1038/nature07205 18650914

[52] Martinez FO Gordon S. The M1 and M2 paradigm of macrophage activation: time for reassessment. F1000Prime Rep (2014) 6:13. doi: 10.12703/P6-13 24669294 PMC3944738

[53] Mosser DM Edwards JP. Exploring the full spectrum of macrophage activation. Nat Rev Immunol (2008) 8(12):958–69. doi: 10.1038/nri2448 PMC2724991 19029990

[54] Barrera MJ Aguilera S Castro I Carvajal P Jara D Molina C. Dysfunctional mitochondria as critical players in the inflammation of autoimmune diseases: potential role in sjögren’s syndrome. Autoimmun Rev (2021) 20(8):102867. doi: 10.1016/j.autrev.2021.102867 34118452

[55] Zhou R Tardivel A Thorens B Choi I Tschopp J. Thioredoxin-interacting protein links oxidative stress to inflammasome activation. Nat Immunol (2010) 11(2):136–40. doi: 10.1038/ni.1831 20023662

[56] Sun Y Li J Xie X Gu F Sui Z Zhang K. Macrophage-osteoclast associations: origin, polarization, and subgroups. Front Immunol (2021) 12:778078. doi: 10.3389/fimmu.2021.778078 34925351 PMC8672114

[57] Faas M Ipseiz N Ackermann J Culemann S Gruneboom A Schroder F. Il-33-induced metabolic reprogramming controls the differentiation of alternatively activated macrophages and the resolution of inflammation. Immunity (2021) 54(11):2531–46 e5. doi: 10.1016/j.immuni.2021.09.010 34644537 PMC7617137

[58] Murray PJ. Macrophage polarization. Annu Rev Physiol (2017) 79:541–66. doi: 10.1146/annurev-physiol-022516-034339 27813830

[59] Wculek SK Dunphy G Heras-Murillo I Mastrangelo A Sancho D. Metabolism of tissue macrophages in homeostasis and pathology. Cell Mol Immunol (2022) 19(3):384–408. doi: 10.1038/s41423-021-00791-9 34876704 PMC8891297

[60] Horuluoglu B Bayik D Kayraklioglu N Goguet E Kaplan MJ Klinman DM. Pam3 supports the generation of M2-like macrophages from lupus patient monocytes and improves disease outcome in murine lupus. J Autoimmun (2019) 99:24–32. doi: 10.1016/j.jaut.2019.01.004 30679006 PMC6462249

[61] Douglas MR Morrison KE Salmon M Buckley CD. Why does inflammation persist: A dominant role for the stromal microenvironment? Expert Rev Mol Med (2002) 4(25):1–18. doi: 10.1017/s1462399402005264 14987384

[62] Tan Z Wang L Li X. Composition and regulation of the immune microenvironment of salivary gland in sjögren’s syndrome. Front Immunol (2022) 13:967304. doi: 10.3389/fimmu.2022.967304 36177010 PMC9513852

[63] Guilliams M Mildner A Yona S. Developmental and functional heterogeneity of monocytes. Immunity (2018) 49(4):595–613. doi: 10.1016/j.immuni.2018.10.005 30332628

[64] Lavin Y Winter D Blecher-Gonen R David E Keren-Shaul H Merad M. Tissue-resident macrophage enhancer landscapes are shaped by the local microenvironment. Cell (2014) 159(6):1312–26. doi: 10.1016/j.cell.2014.11.018 PMC4437213 25480296

[65] Grootveld AK Kyaw W Panova V Lau AWY Ashwin E Seuzaret G. Apoptotic cell fragments locally activate tingible body macrophages in the germinal center. Cell (2023) 186(6):1144–61.e18. doi: 10.1016/j.cell.2023.02.004 36868219 PMC7614509

[66] Peng Y Zhou M Yang H Qu R Qiu Y Hao J. Regulatory mechanism of M1/M2 macrophage polarization in the development of autoimmune diseases. Mediators Inflammation (2023) 2023:8821610. doi: 10.1155/2023/8821610 PMC10270764 37332618

[67] Lu X Li N Zhao L Guo D Yi H Yang L. Human umbilical cord mesenchymal stem cells alleviate ongoing autoimmune dacryoadenitis in rabbits via polarizing macrophages into an anti-inflammatory phenotype. Exp eye Res (2020) 191:107905. doi: 10.1016/j.exer.2019.107905 31891674 PMC8612174

[68] Tzachanis D Berezovskaya A Nadler LM Boussiotis VA. Blockade of B7/cd28 in mixed lymphocyte reaction cultures results in the generation of alternatively activated macrophages, which suppress T-cell responses. Blood (2002) 99(4):1465–73. doi: 10.1182/blood.v99.4.1465 11830501

[69] Pirgova G Chauveau A MacLean AJ Cyster JG Arnon TI. Marginal zone sign-R1(+) macrophages are essential for the maturation of germinal center B cells in the spleen. Proc Natl Acad Sci United States America (2020) 117(22):12295–305. doi: 10.1073/pnas.1921673117 PMC7275705 32424104

[70] Wahren-Herlenius M Dörner T. Immunopathogenic mechanisms of systemic autoimmune disease. Lancet (London England) (2013) 382(9894):819–31. doi: 10.1016/s0140-6736(13)60954-x 23993191

[71] Gottenberg JE Cagnard N Lucchesi C Letourneur F Mistou S Lazure T. Activation of ifn pathways and plasmacytoid dendritic cell recruitment in target organs of primary sjögren’s syndrome. Proc Natl Acad Sci United States America (2006) 103(8):2770–5. doi: 10.1073/pnas.0510837103 PMC1413808 16477017

[72] Manoussakis MN Boiu S Korkolopoulou P Kapsogeorgou EK Kavantzas N Ziakas P. Rates of infiltration by macrophages and dendritic cells and expression of interleukin-18 and interleukin-12 in the chronic inflammatory lesions of sjögren’s syndrome: correlation with certain features of immune hyperactivity and factors associated with high risk of lymphoma development. Arthritis rheumatism (2007) 56(12):3977–88. doi: 10.1002/art.23073 18050195

[73] Vettori S Irace R Riccardi A Iacono D Pellecchia L Vicedomini L. Serum cxcl4 increase in primary sjögren’s syndrome characterizes patients with microvascular involvement and reduced salivary gland infiltration and lymph node involvement. Clin Rheumatol (2016) 35(10):2591–6. doi: 10.1007/s10067-016-3386-7 27562035

[74] Ray PS Fox PL. A post-transcriptional pathway represses monocyte vegf-a expression and angiogenic activity. EMBO J (2007) 26(14):3360–72. doi: 10.1038/sj.emboj.7601774 PMC1933405 17611605

[75] Njock MS Cheng HS Dang LT Nazari-Jahantigh M Lau AC Boudreau E. Endothelial cells suppress monocyte activation through secretion of extracellular vesicles containing antiinflammatory micrornas. Blood (2015) 125(20):3202–12. doi: 10.1182/blood-2014-11-611046 PMC4440888 25838349

[76] Hou X Hong X Ou M Meng S Wang T Liao S. Analysis of gene expression and tcr/B cell receptor profiling of immune cells in primary sjögren’s syndrome by single-cell sequencing. J Immunol (Baltimore Md: 1950) (2022) 209(2):238–49. doi: 10.4049/jimmunol.2100803 35705251

[77] Yang Y Wang Y Guo L Gao W Tang TL Yan M. Interaction between macrophages and ferroptosis. Cell Death Dis (2022) 13(4):355. doi: 10.1038/s41419-022-04775-z 35429990 PMC9013379

[78] Yan J Horng T. Lipid metabolism in regulation of macrophage functions. Trends Cell Biol (2020) 30(12):979–89. doi: 10.1016/j.tcb.2020.09.006 33036870

[79] Cai T Du P Suo L Jiang X Qin Q Song R. High iodine promotes autoimmune thyroid disease by activating hexokinase 3 and inducing polarization of macrophages towards M1. Front Immunol (2022) 13:1009932. doi: 10.3389/fimmu.2022.1009932 36325332 PMC9618622

[80] Tannahill GM Curtis AM Adamik J Palsson-McDermott EM McGettrick AF Goel G. Succinate is an inflammatory signal that induces il-1β through hif-1α. Nature (2013) 496(7444):238–42. doi: 10.1038/nature11986 PMC4031686 23535595

[81] O’Neill LA Pearce EJ. Immunometabolism governs dendritic cell and macrophage function. J Exp Med (2016) 213(1):15–23. doi: 10.1084/jem.20151570 26694970 PMC4710204

[82] Yousefzadeh MJ Flores RR Zhu Y Schmiechen ZC Brooks RW Trussoni CE. An aged immune system drives senescence and ageing of solid organs. Nature (2021) 594(7861):100–5. doi: 10.1038/s41586-021-03547-7 PMC8684299 33981041

