# Supplementary figures and images for: Characterisation of macrophage infiltration and polarisation based on integrated transcriptomic and histological analyses in Primary Sjögren’s syndrome
Source: Front Immunol. 2023 Nov 3;14:1292146. doi: 10.3389/fimmu.2023.1292146 (PMC10656691; doi:10.3389/fimmu.2023.1292146)

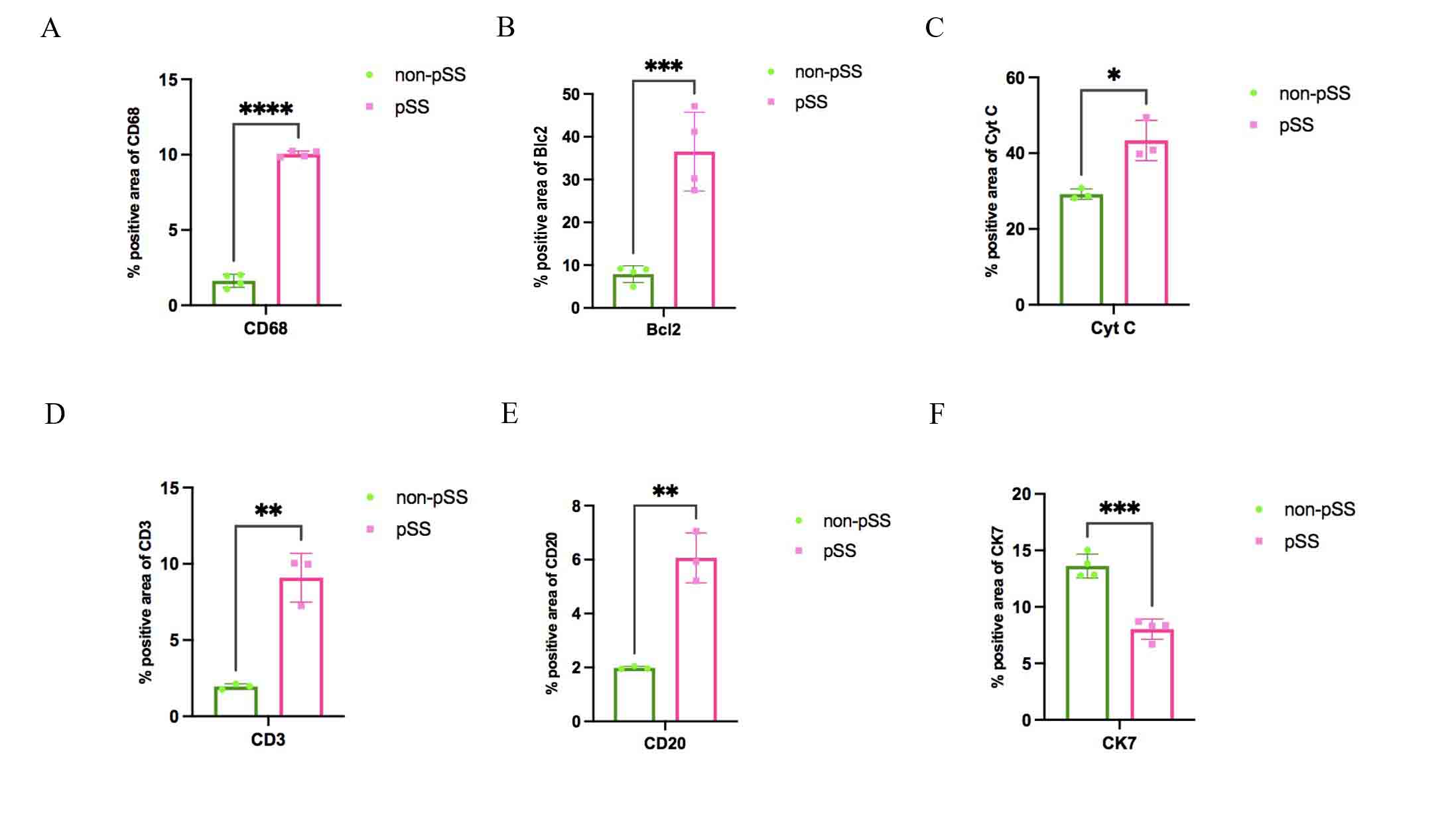

Supplement: Supplementary file 1 [file Image_1.jpg]

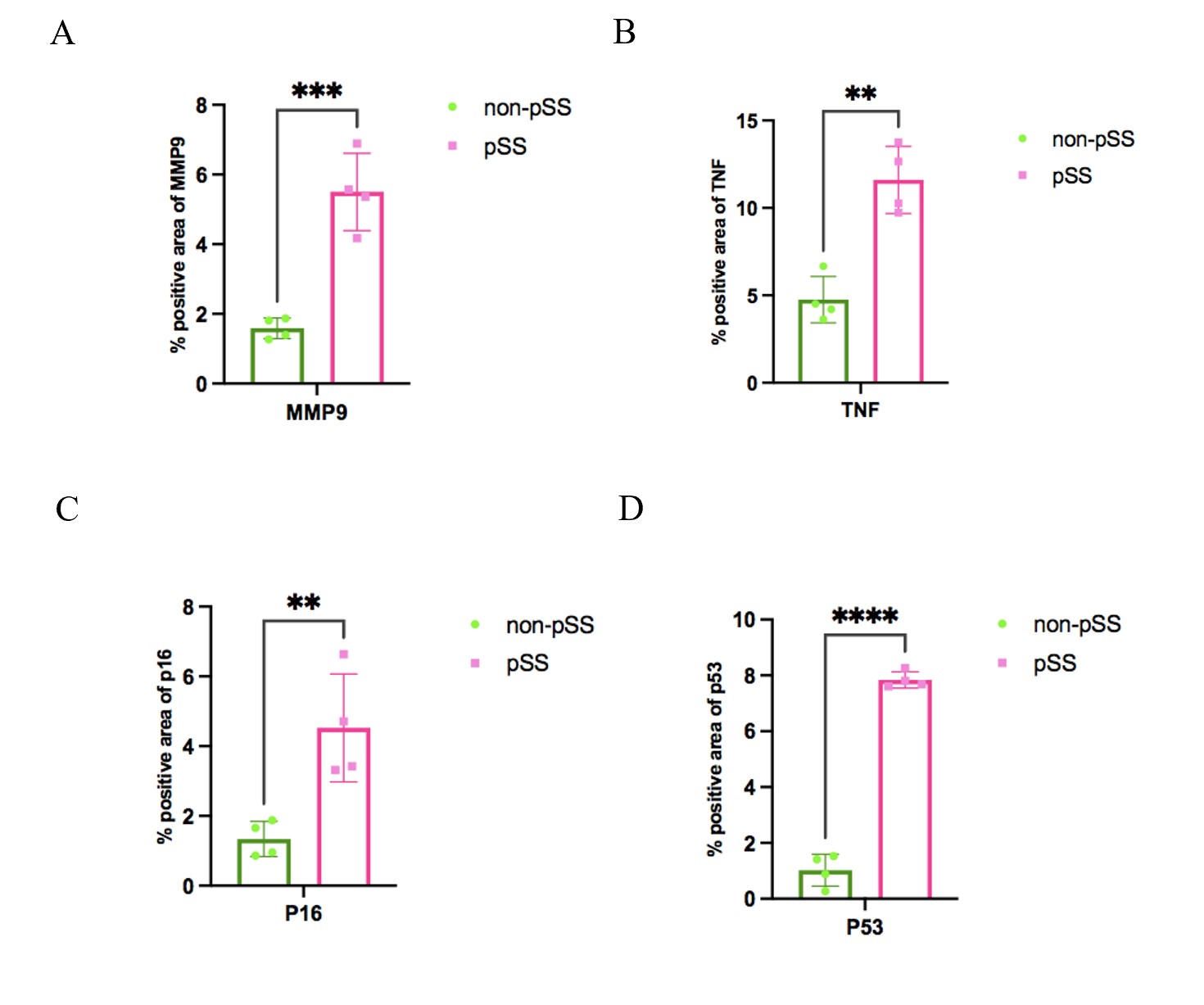

Supplement: Supplementary file 2 [file Image_2.jpeg]
